# Supplementary material for: Understanding the menstrual health self-care practices and experiences among women with physical disabilities in rural Nepal: A qualitative study
Source: PLoS One. 2025 Dec 10;20(12):e0338109. doi: 10.1371/journal.pone.0338109 (PMC12694883; doi:10.1371/journal.pone.0338109)
Supplement: S1 File — (PDF) [file pone.0338109.s001.pdf]

Semi-Structured Interview Guideline: Self-care Practices of Menstruation health and Menstrual Hygiene Management by Married Women with physical disabilities of Karnali Province, Nepal: A Qualitative Study

Introduction

Objective: To explore the different self-care practices related to menstrual health and menstrual hygiene management by married Women with physical disabilities of Karnali Province, Nepal

Interviewer should introduce themselves briefly, mentioning the objectives, rationale, and outcome of the study. After informed consent data should be collected ensuring the participant's confidentiality.

Socio-demographic information.

1. Could you tell me your name, surname, caste, ethnicity, educational status?
2. What is your occupation?
3. Which category of disability do you fall in? (type of cards)

Self-awareness:

1. Do you know what menstruation is? Could you elaborate on it?
2. How much awareness do you have regarding the availability of different kinds of menstrual products (Probe: synthetic pad, moon cups, tampons, cloth pads, period panties)?
3. Do you know how to maintain hygiene during menstruation (Use of synthetic pads, reusable pads, bathing, cleaning the intimate areas, proper disposal of pads)
4. Do you easily get information regarding menstruation? From where do you access the information regarding menstrual health? (Probe: Peers, Elders, teachers, social media, books, magazines)
5. For you, how important is menstrual health and its hygiene management in comparison to other health concerns? And why it is like this? Could you elaborate more?

2. Self-Management

1. As often how do you treat your menstrual cramps if required? (Use of medicines, herbal medicines, use of hot bags, etc) and what is its effectiveness?
2. What kind of home remedies do you follow to manage your menstrual pain (use of hot water, honey water, etc)? And how did you learn them?
3. Do you practice self-massage like: oiling your abdomen with warm oils? Have you tried this before? If yes can you describe its benefits to you?
4. What kind of food do you consume during your period? Why do you consume them?
5. Do you restrict any kind of food consumption during your period cycle? If yes, could you provide me with the reasons?
6. How often do you visit professional health services for severe menstrual pain and issues related to it? Why do you do so? Could you elaborate on it?
7. How do you calculate to predict your next period cycle (Probe: calendar, gut feeling, asking somebody, self-remembering) From where did you learn about this?

8. What kinds of menstrual products do you use and How do you decide to use any one of them? (Probe: accessibility, availability, affordability)
9. How do you usually dispose of your pads (Probe: disposing of it in the dustbin, throwing it on the river, burning it, etc)
10. What kinds of traditional practices do you practice for your overall menstrual health management (Probe: Seclusion during menstruation, not doing certain things, not eating certain foods, etc)
11. How much do you find home remedies reliable over allopathic medicines? Why do you think so?
12. How do you seek help for menstrual issues? (Probe: asking family members, friends, health professionals, FCHVs, etc) Who helps you normally?
13. How do you manage your mood swings, and stress during your menstrual cycle? (Probe: talking to a friend, doing yoga, meditation, talking to family members, seeking professional help)

Self-Testing

1. In case of absence of menstruation, do you use a pregnancy kit? How reliable have you found it?
2. How often do you observe your menstrual blood regarding its consistency, color, odor, and volume? And why do you do it?

Facilitators and Barriers to Self-Care in Menstrual Practices

1. What knowledge and skills do you have that help you fight against menstrual taboos? How has it contributed positively to your life? From where did you acquire it (Probe: Friends, Family members, FCHVs, Workshops, training, health professionals, news, digital platforms, radio, T.V, social media)

2. Who are your support systems? (Friends, Family members, FCHVs, health professionals), How has it impacted you? (Probe: easy to fight against taboo, easily manage menstrual hygiene)
3. How has been your experience with healthcare professionals regarding menstrual health? (Probe: attitudinal barrier due to disability, empathetic, no discrimination)
4. So what do you think regarding the empowerment of women with physical disabilities or any kind of disabilities? How can this facilitate improved self-care practices of menstrual health and hygiene management?
5. What do you think being a woman impacts decision-making practices to adopt self-care practices or not? If yes then how does it happen? (Gender roles: reproductive, submissive roles)
6. Do you think the issues regarding menstrual health and its hygiene management are adequately prioritized in your house, community, and even in the nation in comparison to other health issues like Diabetes, Communicable diseases, etc?

7. According to you, how can we foster the self-care practices of menstruation health and its hygiene management among women with disabilities?
8. Is there anything you want to add? Please feel free to express anything.

Thank you very much for your time and patience.
