# Supplementary material for: Understanding the menstrual health self-care practices and experiences among women with physical disabilities in rural Nepal: A qualitative study
Source: PLoS One. 2025 Dec 10;20(12):e0338109. doi: 10.1371/journal.pone.0338109 (PMC12694883; doi:10.1371/journal.pone.0338109)
Supplement: S2 File — (PDF) [file pone.0338109.s002.pdf]

Self-care Practices of Menstruation Hygiene Management by Married Women with physical disabilities of Karnali Province, Nepal: A Qualitative Study

Information sheet:

This qualitative study will be conducted among married women with physical disabilities of Karnali province to gain insights regarding self-care practices for menstrual health and hygiene management. By exploring the situation, its facilitators, and barriers, this study aims to build evidence for advocacy and policy implementation regarding self-care practices for menstrual health and hygiene management in Nepal. The study requires participants from (18-49) years of age. The data collection will be conducted between September 1st to October

Consent Sheet:

Namaste! My name is ________________ and I am collecting data for study/research with the objective that I stated above. I would like to know your overall self-care practices regarding menstruation hygiene, management with its facilitators, and Barriers. For this, I have to take an interview with you. The interview may take 30 – 45 minutes and I will ask about your knowledge and experiences regarding self-awareness, self-management, and self-testing as well as the types of self-care menstrual health practices you adopt during your menstruation for its overall management. I would be very grateful if you could spend some time talking with me. These discussions will be audio-recorded for accuracy and researcher bias. I will maintain your anonymity and everything you tell me will be kept strictly confidential. You will be asked to provide basic demographic information such as age, marital status, educational background, and any specific visual impairments you have but none of this will be disclosed for any reason. Your identities will be pseudonymized to maintain confidentiality. Since your participation is voluntary, you can choose not to answer at any time you like. For participation, there is no monetary benefit associated with the study and there is no known harm associated with the study too. The organization has an in-house counselor and medical officers in the link who are in need and will provide counseling and medical checkups if you feel stressed, feel emotional, or get sick. Participating in this study may not have immediate direct benefits for you. However, your insights and experiences will contribute to a greater understanding of the self-care challenges faced by women with disabilities which can impact policy implementation as well. The researcher will provide a supportive environment and ensure your emotional well-being throughout the process. If you have any questions, or concerns, or wish to obtain additional information about the study, please feel free to contact the researchers at the following contact details:

Researcher Name:

Contact Number:

Contact Email:

Ethical review board/ Nepal health research council:

Contact number: 977-1-4254220

Email: nhrc@nhrc.gov.np

Informed consent

Do I have your permission to continue?

Yes: ☐ Respondent agrees to be interviewed and then starts the interview.

No: ☐ Respondent does not agree to be interviewed then terminate the interview.

Signature of Interviewer: .... Date: ....

Interview start time: .... End time: ....
