# Supplementary material for: Understanding the menstrual health self-care practices and experiences among women with physical disabilities in rural Nepal: A qualitative study
Source: PLoS One. 2025 Dec 10;20(12):e0338109. doi: 10.1371/journal.pone.0338109 (PMC12694883; doi:10.1371/journal.pone.0338109)
Supplement: Attachment — Submitted filename: Adhikari 2024 paper review 1.docx [file pone.0338109.s003.docx]

The paper is written about a topic still emerging in current scientific discourse, so it is a valuable addition. However, some areas could be strengthened to ensure it reflects the existing evidence. For instance, the authors would benefit from drawing on existing data about disability and menstrual health in Nepal and other countries in their discussion. I have written detailed comments against each section below, as well as general comments at the end.

**Abstract**

- Include the sampling methods used and sample size.
- Some sentences are long and therefore a bit confusing (e.g. ‘Women with physical disabilities self-managed their menstrual cramps with home remedies and medicines and used clothes as a pad to manage the menstruation flow, and self-tested themselves using pregnancy kit in case of missing period to diagnose the pregnancy and relied on their body response and symptoms to self-tracking of menstrual cycle while observed blood flow to self-diagnose menorrhagia’ in the abstract. I suggest getting someone else to proof-read your paper or use AI to improve readability

**Introduction**

- Include a definition of menstrual health (or menstrual health and hygiene). This is a commonly used definition, which includes hygiene: Hennegan J, Winkler I, Bobel C, Keiser J, Hampton J, Larsson G, et al. Menstrual health: a definition for policy, practice, and research. Sexual and reproductive health matters. 2021;29. doi: 10.1080/26410397.2021.1911618.
- Give more information about disability in Nepal, including the population living with disabilities, those with physical disabilities (if available) and social support/protection provided by the govt and NGOs. This covers the disability cards which you refer in the manuscript. Also include information on the GoN and CSO’s approaches to improving the menstrual health of women and girls as these are considerable
- State the research aim more explicitly and include your research questions or objectives

**Methods**

More detail is required to ensure other researchers could replicate your research design and methods. Below are specifics.

- Include a reference for Braun and Clarke, line 119, and give a brief explanation of this approach
- Line 128 – who compiled the list? The authors with input from the OPDs or just the latter?
- Include your definition of ‘physical disability.’ How did you determine this in your study (e.g. self-reported or from medical lists)?
- Line 137 – explain/justify why you selected IDIs and FGDs as methods.
- Line 138-140: I find this a bit confusing- ‘with 10 participants were conducted utilizing the semi-structured interview guidelines to gather contextually relevant insights from women with physical disabilities‘. It sounds like the 10 participants used the topic guide themselves, which I don’t think you mean. Instead, could you fully explain the development and testing of the topic guides (including the themes of interest), before stating the number of participants? After that, explain the FGD process – who led them, what topics were covered. Then state the number of participants involved in the FGSs.
- Line 140 – explain what definition you are using for ‘reproductive age’ and what the age range is
- Line 142 – was the informed consent process and interview conducted in Nepali? Who were the interviewers and what are their backgrounds? How does this contribute to the research? Where were the interviews and FDGs conducted? Why did you take verbal and not written consent? Was this witnessed?
- Line 143 – did you do an audio or visual recording? If the former, write ‘audio recording’
- Please add your topic guides as supporting information files
- Include a description of any decision made to stop data collection (ie reached data saturation) and justify this
- Please describe the Maintenance/Preservation of subject anonymity and confidentiality.
- Table 1. Participants could be identified through these details (eg we know that they are women with physical disabilities and can tell the severity of the disability because of the colour card, living in a specific area and of a certain age) so the reader can work out who said what in the results. I suggest changing the table so you aggregate the number of people across the different colour card but don’t include the participant number. This applies for the IDIs and FGs
- **Data processing and analysis:** Please include how you identified themes and concepts. E.g. did you start with a framework or codebook or did you generate themes and concepts from your data? Also detail who analysed the data. If more than one person coded the data, what process did you follow to resolve any disagreements? Some of this information is covered in the results, but I’d suggest moving line 159-166 to your data analysis section instead.

**Results**

- Ensure you write the results in past tense. E.g. line 210 should be ‘threw them outside’, not ‘throw.’
- Line 200. Can you start by listing all the menstrual materials used and then use the terms consistently throughout? E.g. line 207 – does synthetic pads mean commercially produced pads? If so, that might be a clearer term to use. What do you mean by ‘homemade’ pad? Are these menstrual cloths (ripped sari) or are they tailor made reusable pads? Giving more detail at the start before going into the who uses what in which area would be helpful.
- Line 218 – does this refer to tracking the menstrual cycle? If so, please state that.
- Line 219-220 is about pregnancy testing, but I’d suggest discussing that after the results around tracking the mens cycle (presumably they are tracking their mens cycle and know that their period is late and that is why they are taking pregnancy tests?). I’m also unclear what you mean by ‘the majority of them were found not to practice it’ – what does ‘it’ refer to?
- Line 234: should ‘blood. To know’ be one sentence?
- Line 236: ‘one of the participants’ is unfinished. Should you end the sentence with ‘stated’, ‘expressed’ and then go into the quote?
- Line 247: ‘participants knew about the definition of mens’. Explain what this and refer back to a definition given in the intro (see my comment above)
- Line 274. Give an example of an ‘accessible mobile app’. In line 367-370 you refer to facebook. Can you discuss these topics together or cross reference the sections to allow the reader to understand how the sections relate to each other?
- You report that some people got information from ‘course books’. Can you state earlier how many participants were in school? Then, in this section state where they access these course books – at school in healthcare centres? This is important because existing evidence states that children with disabilities are more likely to be out of school than those without disabilities, so relying on schools to disseminate menstrual health information is not sufficient.
- Line 326 – you are discussing menstrual materials used here, but you have spoken about it earlier on. Consider merging the sections

**Discussion**

- Line 416. I’m afraid this isn’t the first paper that explores the issues. These two papers explore disability and menstrual health, which includes physical disability and self-care practices: <https://bmcpublichealth.biomedcentral.com/articles/10.1186/s12889-021-10439-y> and <https://equityhealthj.biomedcentral.com/articles/10.1186/s12939-021-01463-w>. There is also a systematic review from 2019 that looked at this issue across LMICs (<https://journals.plos.org/plosone/article?id=10.1371/journal.pone.0210974>). Please amend your first sentence. Please review a broader range of literature on disability and menstrual health for inclusion in your discussion as most of it either focuses on menstrual health (without considering disability) or disability (without considering menstrual health). Quite a lot of such evidence exists and should be reflected in your discussion for greater relevance.
- Line 422 – You are stating what your results cover so I don’t understand what reference 2 relates to?
- When you discuss existing literature in relation to your findings, include more detail so the reader understands the existing literature without having to read the paper. E.g. line 428 – what rural settings and in what countries? You do this well in line 436-438.
- Line 475-477 – these platforms and chat services must be referenced.
- Line 496: See this policy analysis of Nepal’s menstrual health and WASH policies to understand if/how disability is included compared to gender. This is a very relevant paper for your point here: <https://equityhealthj.biomedcentral.com/articles/10.1186/s12939-021-01463-w>
- Include a discussion on the transferability of research findings to other settings
- Line 515. Have a separate section on the study’s strengths and limitations
- Reflect on the influence of the researcher[s] on data, including a consideration of how the researcher(s) may have introduced bias to the results is included

**General**

- Double check your spelling. For example, you refer to ‘clothes’ instead of ‘cloths’ several times in the manuscript
- Before submitting your next draft, double-check the grammar as there are a couple of errors. E.g. Line 209 and 218 are missing full stops. Also check the use of capitals within sentences. E.g. line 257 – Likewise, Despite…
- Explain all acronyms when they are first used (eg line 222)
- Write words in full. E.g. don’t use terms like didn’t, couldn’t, etc or starting a sentence with ‘because’. These are too informal for a manuscript.
