# Supplementary material for: Understanding the menstrual health self-care practices and experiences among women with physical disabilities in rural Nepal: A qualitative study
Source: PLoS One. 2025 Dec 10;20(12):e0338109. doi: 10.1371/journal.pone.0338109 (PMC12694883; doi:10.1371/journal.pone.0338109)
Supplement: Attachment — Submitted filename: Response to Reviewer.docx [file pone.0338109.s005.docx]

Editor’s/Reviewer’s comments

Response

Reviewer # 1

**Abstract**

1. Include the sampling methods used and sample size.

**Answer:** Dear Reviewer, thank you very much for your feedback. Purposive sampling was used in the study. For the data collection. 21 In-depth Interviews (IDI) and 1Focus Group Discussion (FGD) with a total 31 married women with physical disabilities aged 18-49 years living in Karnali Province until data saturation was reached. This also has been revised and incorporated in line number Line 31-33 in clean version of manuscript.

2. Some sentences are long and therefore a bit confusing (e.g. ‘Women with physical disabilities self-managed their menstrual cramps with home remedies and medicines and used clothes as a pad to manage the menstruation flow, and self-tested themselves using pregnancy kit in case of missing period to diagnose the pregnancy and relied on their body response and symptoms to self-tracking of menstrual cycle while observed blood flow to self-diagnose menorrhagia’ in the abstract. I suggest getting someone else to proof-read your paper or use AI to improve readability

**Answer:** Thank you so much for your constructive feedback. The comment has been addressed in the manuscript line number 38-50 in clean version of Manuscript.

**Introduction**

3. Include a definition of menstrual health (or menstrual health and hygiene). This is a commonly used definition, which includes hygiene: Hennegan J, Winkler I, Bobel C,Keiser J, Hampton J, Larsson G, et al. Menstrual health: a definition for policy, practice, and research. Sexual and reproductive health matters. 2021;29. doi:10.1080/26410397.2021.1911618.

**Answer:** Thank you so much for the suggestion. The definition of the menstrual hygiene has been added in the manuscript line number 73-80 in clean version of Manuscript.

4. Give more information about disability in Nepal, including the population living with disabilities, those with physical disabilities (if available) and social support/protection provided by the govt and NGOs. This covers the disability cards which you refer in the manuscript. Also include information on the GoN and CSO’s approaches to improving the menstrual health of women and girls as these are considerable

**Answer:** Thank you so much for the suggestion, the information has been incorporated in the manuscript. Line number 84 to 101 in clean version of Manuscript.

5. State the research aim more explicitly and include your research questions or objectives

**Answer:** Thank you for pointing this out. This has been reviewed and addressed in the line 116-122 in clean version of Manuscript.

6. Line 128 – who compiled the list? The authors with input from the OPDs or just the latter?

**Answer:** Thank you for the query. The organization DAARDO, BWAN and BYAN provided the list of participants. Those participants who provided the consent for interviews were listed down. Finally, the principal investigator compiled the list. This has been explained further in the line 170-132 to 139 in clean version of Manuscript.

7. Include your definition of ‘physical disability.’ How did you determine this in your study (e.g. self-reported or from medical lists)?

**Answer:** The definition of the physical disability as per the government of Nepal is incorporated in line84-93 of clean version of manuscript. The Nepal Census 2078 has reported that 2.2% of the population has a disability, with 36.75% of those having physical disabilities that entails the highest number of disabilities to other form of disabilities.

8. Line 137 – explain/justify why you selected IDIs and FGDs as methods.

**Answer:** Thank you for the query. Since this study adopts the qualitative method approach, detailed qualitative information on people's thoughts, feelings, experiences, and behaviors can be collected by IDI and FGD. Specifically, to collect detailed, personalized experiences from individual participants without any external influence IDI was conducted while to gather a range of opinions, shared beliefs, collective attitude, gathering insights from multiple homogenous participants at once FGD was conducted. This has been described in the line 159 to 162 of clean version of manuscript.

9. Line 138-140: I find this a bit confusing- ‘with 10 participants were conducted utilizing

the semi-structured interview guidelines to gather contextually relevant insights from women with physical disabilities ‘. It sounds like the 10 participants used the topic guide themselves, which I don’t think you mean. Instead, could you fully explain the development and testing of the topic guides (including the themes of interest), before stating the number of participants? After that, explain the FGD process – who led them, what topics were covered. Then state the number of participants involved in the FGDs

**Answer:** Thank you for flagging this. This has now been reviewed and revised in the line 144 to 151 in the clean version of manuscript.

10. Line 140 – explain what definition you are using for ‘reproductive age’ and what the age range is

**Answer:** Thank you very much for the query. The used definition of reproductive health is of the World Health Organization which defines reproductive age group as those between the ages of 15-49.

11. -	Line 142 – was the informed consent process and interview conducted in Nepali? Who were the interviewers and what are their backgrounds? How does this contribute to the research? Where were the interviews and FDGs conducted? Why did you take verbal and not written consent? Was this witnessed?

**Answer:** Thank you for your query. Yes, the informed consent process and interview was conducted in Nepali. IDIs were taken at their respective residence and FGD was taken in the meeting hall. The participants had physical disabilities and some of them were not able to provide written consent therefore in order to maintain the homogeneity with informed consent, verbal consent was taken. Yes, this was witnessed by enumerator and participant. The explanation is incorporated in line 152 to 159 in clean version of Manuscript.

12. Line 143 – did you do an audio or visual recording? If the former, write ‘audio recording’

**Answer:** Thank you for your comment, audio radio was employed which is incorporated in line 156 in clean version of manuscript.

13. Please add your topic guides as supporting information files

**Answer:** The semi-structured interview guideline has been uploaded as supplementary file.

14. Include a description of any decision made to stop data collection (ie reached data saturation) and justify this

**Answer:** In this qualitative study, we employed purposive sampling to select participants who could provide rich insights and their experiences relevant to our research objectives. Data collection was stopped upon reaching data saturation (as guided by the Fusch. Et. al)—the point at which no new themes or significant insights were emerging from additional interviews or discussion. This decision was made to ensure that the collected data fully captured the diversity of perspectives while avoiding redundancy. By monitoring for recurring patterns across responses, we confirmed that further data would not contribute additional value. By identifying data saturation, we decided to stop the data collection affirming that the sample size was sufficient for comprehensive analysis. Incorporated in line 160 to 166 in clean version of manuscript.

15. Please describe the Maintenance/Preservation of subject anonymity and confidentiality.

**Answer:** In order to maintain the confidentiality and anonymity of the participants, the researchers have assigned the participant ID number. removing identifying information and using coded identifiers in place of actual names. The actual data can be accessed by PI and Co-PI only. The document is uploaded in google drive and only the PI has access to an edit option while it requires permission to other research members to access edit option of the data. The data is kept in password-protected word document and only those who have access can view it. This is incorporated from line 192-197.

16. Table 1. Participants could be identified through these details (e.g. we know that they are women with physical disabilities and can tell the severity of the disability because of the color card, living in a specific area and of a certain age) so the reader can work out who said what in the results. I suggest changing the table so you aggregate the number of people across the different color card but don’t include the participant number. This applies for the IDIs and FDGs

**Answer:** Thank you for your feedback. We have removed the name of municipality and card color to anonymize the data. Also, we have removed the table and had written in the aggregated form. Since, we have not provided the specific locality for everyone, it is hard to identify the participants. It is incorporated from line 170 to 174 of clean version of manuscript.

17. **Data processing and analysis:** Please include how you identified themes and concepts. E.g. did you start with a framework or codebook, or did you generate themes and concepts from your data? Also detail who analyzed the data. If more than one person coded the data, what process did you follow to resolve any disagreements? Some of this information is covered in the results, but I’d suggest moving line 159-166 to your data analysis section instead.

**Answer:** -Thank you for the query, the data was analyzed using an inductive thematic analysis approach, allowing themes and concepts to emerge directly from the data. This approach was chosen to ensure that the analysis was grounded in participants' perspectives and experiences, rather than imposing predefined categories or frameworks. No initial codebook or theoretical framework was used in coding; instead, we generated themes iteratively from the raw data. (184-191 of the clean version of manuscript.

**Process of Coding and Theme Development**

The data was initially coded by the principal investigator and first co-investigator. The coding process involved close reading and re-reading of the transcripts, during which initial codes were assigned to capture significant aspects of the data relevant to our research query. These codes were then refined, grouped, and organized into potential themes based on patterns and recurring concepts observed across the dataset.

**Coding Consistency and Resolving Disagreements:** Both researchers independently coded a subset of the data to ensure a diverse perspective on the themes and concepts. After coding independently, we met to discuss and compare their codes, identifying any areas of divergence. Disagreements were resolved through discussion and, when necessary, by involving a third researcher to ensure consistency in interpretation. This collaborative approach enhanced the reliability and depth of the thematic analysis, ensuring that all major themes accurately reflected participants' views.

18. Ensure you write the results in past tense. E.g. line 210 should be ‘threw them outside’, not ‘throw.’

**Answer:** Thank you for pointing this out, all the grammars has been thoroughly checked and addressed.

19. Line 200. Can you start by listing all the menstrual materials used and then use the terms consistently throughout? E.g. line 207 – does synthetic pads mean commercially produced pads? If so, that might be a clearer term to use. What do you mean by ‘homemade’ pad? Are these menstrual cloths (ripped sari) or are they tailor made reusable pads? Giving more detail at the start before going into the who uses what in which area would be helpful.

**Answer:** Thank you for the query and we have addressed this in the manuscript line 241 and 242 of clean version of the manuscript. The menstrual materials used were:

1. Homemade pads (Ripped saari, small piece of clothes)
2. Commercially produced pads. (Synthetic menstrual pads)
3. Menstrual underwear
20. Line 218 – does this refer to tracking the menstrual cycle? If so, please state that.

**Answer:** Thank you for flagging this, it is documented in line 300-306 in the clean version of Manuscript.

21. Line 219-220 is about pregnancy testing, but I’d suggest discussing that after the results around tracking the mens cycle (presumably they are tracking their mens cycle and know that their period is late and that is why they are taking pregnancy tests?). I’m also unclear what you mean by ‘the majority of them were found not to practice it’ – what does ‘it’ refer to?

**Answer:** Thank you for your constructive insights, it has been revised accordingly. The pregnancy test in case of missing period is kept after the description of tracking menstrual cycle. Incorporated in Line: 300 to 310 in clean version of manuscript.

22. Line 234: should ‘blood. To know’ be one sentence?

**Answer:** The sentence structure and the grammar has been thoroughly checked and addressed.

23. Line 236: ‘one of the participants’ is unfinished. Should you end the sentence with ‘stated’, ‘expressed’ and then go into the quote?

**Answer:** Thank you for the feedback. It has been revised as per the suggestion.

24. Line 247: ‘participants knew about the definition of mens’. Explain what this and refer back to a definition given in the intro (see my comment above)

**Answer:** Thank you for the feedback. The definition in this sentence refers to the literal meaning of menstruation which is the bleeding occurrence of every month. I have revised accordingly in the manuscript. Line**:** 333 in clean version of manuscript.

25. Line 274. Give an example of an ‘accessible mobile app’. In line 367-370 you refer to facebook. Can you discuss these topics together or cross reference the sections to allow the reader to understand how the sections relate to each other?

**Answer:** Thank you for your query**.** An example of an accessible mobile app is Facebook, which has incorporated several features to ensure usability for individuals with disabilities. These include screen reader compatibility, voice commands, and customizable text size. we mentioned facebook in the context of its accessibility features, and it's relevant here to highlight how such design considerations enhance the app's usability for all users, including those with visual impairments.Another example on menstrual hygiene management includes Pahunch mobile app, hamiform SRHR application which provides comprehensive information and accessible features to access information. The reference is incorporated in line:377

26. You report that some people got information from ‘course books.’ Can you state earlier how many participants were in school? Then, in this section state where they access these course books – at school in healthcare centers? This is important because existing evidence states that children with disabilities are more likely to be out of school than those without disabilities, so relying on schools to disseminate menstrual health information is not sufficient.

**Answer:** Thank you for pointing this out. Unfortunately, we do not have information on this and thus it has not been included in the manuscript.

27. Line 326 – you are discussing menstrual materials used here, but you have spoken about it earlier on. Consider merging the sections

**Answer:** Thank you for the feedback, It has been incorporated accordingly from line 251 to 260.

**Discussion**

28. Line 416. I’m afraid this isn’t the first paper that explores the issues. These two papers explore disability and menstrual health, which includes physical disability and self-care practices: https://bmcpublichealth.biomedcentral.com/articles/10.1186/s12889-021-10439-y and https://equityhealthj.biomedcentral.com/articles/10.1186/s12939-021-01463-w. There is also a systematic review from 2019 that looked at this issue across LMICs (https://journals.plos.org/plosone/article?id=10.1371/journal.pone.0210974). Please amend your first sentence. Please review a broader range of literature on disability and menstrual health for inclusion in your discussion as most of it either focuses on menstrual health (without considering disability) or disability (without considering menstrual health). Quite a lot of such evidence exists and should be reflected in your discussion for greater relevance.

**Answer:** Thank you for flagging this, the first sentence of the discussion has been removed. The clear definition of self-care is not well established in the articles shared by the reviewer. This article looks across all the dimension of self-care as identified by the WHO providing comprehensive analysis of self-care in MHM among women living with physical disability. We have also revisited the discussion section, edited, and revised the references.

29. Line 422 – You are stating what your results cover so I don’t understand what reference 2 relates to?

**Answer:** Thank you for pointing out the error, it has been removed in the manuscript.

30. When you discuss existing literature in relation to your findings, include more detail so the reader understands the existing literature without having to read the paper. E.g. line 428 – what rural settings and in what countries? You do this well in line 436-438.

Answer: This has been well explained and revised now in relevant lines of the discussion section. Line:538 in the clean version of Manuscript.

31. Line 475-477 – these platforms and chat services must be referenced.

**Answer:** Thank you for flagging this. It has been addressed accordingly in the line 387.

32. Line 496: See this policy analysis of Nepal’s menstrual health and WASH policies to understand if/how disability is included compared to gender. This is a very relevant paper for your point here: https://equityhealthj.biomedcentral.com/articles/10.1186/s12939-021-01463-w

**Answer:** Thank you for the suggestion. It has been incorporated in the line 614 to 617.

33. Include a discussion on the transferability of research findings to other settings

**Answer:** Thank you for pointing this out. This has been mentioned in line 634-643.

34. Line 515. Have a separate section on the study’s strengths and limitations

**Answer:**	Thank you for your feedback. This has been added in the line 626 to 644 in the revised version of manuscript.

35. Reflect on the influence of the researcher[s] on data, including a consideration of how the researcher(s) may have introduced bias to the results is included

**Answer:**	Thank you for your suggestion**.**This has been illustrated and well explained in the line 631 to 634

**General**

36. Double check your spelling. For example, you refer to ‘clothes’ instead of ‘cloths’ several times in the manuscript

**Answer**: Thank you for the suggestion, we have thoroughly reviewed the language edit and addressed in the manuscript.

37. Before submitting your next draft, double-check the grammar as there are a couple of errors. E.g. Line 209 and 218 are missing full stops. Also check the use of capitals within sentences. E.g. line 257 – Likewise, Despite…

**Answer:** Thank you for pointing out the errors. All the grammar has been thoroughly checked and corrected as per the feedback.

38. Explain all acronyms when they are first used (e.g. line 222)

**Answer:** Thank you for the comment, The manuscript has been revised accordingly.

39. Write words in full. E.g. don’t use terms like didn’t, couldn’t, etc. or starting a sentence with ‘because’. These are too informal for a manuscript.

**Answer:** Well noted. The manuscript has been revised accordingly.

Reviewer #2

1. I suggest instead of “disabled” and “women with physical disability”, the authors should use “differently abled” because "differently abled" reflects a more respectful, inclusive, and optimistic approach to a person with limited mobility, emphasizing their potential and unique capabilities. It aligns with the concept of "person-first language," which focuses on the person rather than their condition. This approach promotes the idea that an individual's identity is not defined by their physical conditions.

Ans: Thank you for the comment. The United Nations Convention on the Rights of Persons with Disabilities (UNCRPD) uses "persons with disabilities. UNCRPD is a global human rights treaty focused on safeguarding the rights and dignity of persons with disabilities. Established in 2006, it provides a guideline for countries to acknowledge the diversity, autonomy, and equality of individuals with disabilities, supporting their active participation in society. The convention promotes the term "persons with disabilities," emphasizing that every individual is differently abled irrespective to their disabilities and disabilities are a natural part of the human experience and do not limit a person’s unique abilities or potential.

2. Why is referencing not done for Braun and Clarke in the references?

**Answer:** Thank you for your insightful comment. The comment is addressed in the manuscript. Line-182 of clean version of the manuscript.

3. How 21 in-depth interviews and 1 FGD were determined as the sample?

**Answer:** Thank you for the query. We did not determine the sample size to begin with and adopted purposive sampling to identify individuals who have specific experiences or characteristics which will support in generating rich data to fulfill the research objective. The data was collected until the data saturation was obtained as identified by Fush et. al and the data saturation was obtained with the 21^st^ IDI and the FGD conducted meaning that additional interviews and discussions were not yielding new information or themes which indicated that the responses was repetitive. This has been explained in the line 160 to 166 of clean version of manuscript.

4.How were the three organizations, namely DARRDO, BWAN, and BYAN, incorporated into the sampling process? Please explain their roles. BWAN and BYAN are associations for visually challenged persons; it is highly likely that your data mostly represent visually challenged persons while not the other types of physically challenged persons?

**Answer:** Our contact organization with whom we had strong professional relationships were DAARDO, BWAN and BYAN. They have good networking with other organizations and persons with physical disability. These organizations helped us to link with eligible participants with physical disability for this study. This has also been explained in the line 133-135 of the clean version of manuscript.

5. What are those severities and yellow & blue cards mentioned in Table 1? The manuscript does not explain how the type of disability can be understood using those terminologies.

**Answer:** Thank you for the query. The Government of Nepal categorizes disability into four levels based on severity, each associated with a specific color-coded disability card:

**Profound Disability** - red disability card, **Severe Disability** - blue disability card, **Moderate Disability** - yellow disability card, **Mild Disability** - white disability card. To avoid any form of confusion, the table has been removed.

6. Authors should mention how the identities of participants are anonymized. Who can access the data?

**Answer:** Thank you for the comment. In order to maintain the confidentiality and anonymity of the participants, the researchers have assigned the participant ID number. removing identifying information and using coded identifiers in place of actual names. The actual data can be accessed by PI and Co-PI only. The document is uploaded in google drive and only the PI has access to an edit option while it requires permission to other research members to access edit option of the data. The data is kept in password-protected word document and only those who have access can view it. This has been incorporated into the line 192 to 197 in the clean version of manuscript.

7. What are CBOs?

**Answer:** Thank you for the query. CBOs is the short form for community-based organization in line 445 of clean version of manuscript. We have used full form in the manuscript for the first time and used the abbreviation later on.

8.The full form of FCHV should come when it is first used in the manuscript, i.e. in the abstract.

**Answer:** Thank you very much, it is well noted and revised accordingly in the manuscript in line 598 The full form of FCHV is Female community Health Volunteer.

9. One of the participants.” before quotes is grammatically incorrect. Authors may write “One of the participants said” or “According to one of the participants;”

**Answer:** Thank you for the comment. All the grammars in the manuscript have been thoroughly checked and addressed.

10. The authors confined the study to differently abled married women, but they failed to capture the experiences that are unique to this group. Their result is general and not focused on the nature of the group studied. They can at least add two themes to the result, discussing the experiences that are unique to differently abled married women regarding menstruation.

**Answer:** There are two major scope of findings: 1) the women with disabilities have usual needs and self-care practices as other women while 2) the article presents the focused experiences through codes such help-seeking behaviors, empowerment of women with physical disabilities, difficulties in self-medication, body responses to self-monitoring of menstruation, hardship in menstrual hygiene management, exclusion of women with disability from sensitization/awareness session, which are all unique experiences that women with physical disability have experienced. These findings have been mainstreamed in balanced way in each theme and sub-theme. Thus, this paper adequately captures unique findings in the result section for under researched area of menstrual hygiene management and self-care in rural area of Nepal. We have revised few sections to highlight such findings in the paper It is explained in the line 527 to 532, 542 to545.

11.In the discussion section, various issues related to differently abled persons’ challenges that are not found in the results section, such as mental health, diseases, and disorders.

**Answer:** Thank you very much for flagging this. The information has been added in the result section, which was missing. Thank you for this important observation which we missed. It has been incorporated from line 417 to 427 in clean version of Manuscript.
