# Supplementary material for: Understanding the menstrual health self-care practices and experiences among women with physical disabilities in rural Nepal: A qualitative study
Source: PLoS One. 2025 Dec 10;20(12):e0338109. doi: 10.1371/journal.pone.0338109 (PMC12694883; doi:10.1371/journal.pone.0338109)
Supplement: Attachment — Submitted filename: Response_to_Reviewer_auresp_2.docx [file pone.0338109.s006.docx]

Response to Reviewer:

Reviewer #1:

**Abstract**

1. Methods: include your data analysis approach (eg, thematic analysis)

Answer: Thank you very much for your comment. We have incorporated it accordingly. Clean version line: 31 and 32.

2. Result: Avoid using ‘etc’ in academic writing. Some results need more detail. You’re assuming the reader has prior knowledge of the topic, which mightn’t be true. If giving more detail this pushes you over your word count, reduce the number of results. E.g. Line 40 – can you give more details as the reader mightn’t understand the perceived cause and effect. Line 43-44: ‘feeling dependent and vulnerable’. There is a lot to unpack here. Line 48: ‘self-awareness’ or ‘knowledge’?

Answer: Thank you for the comment and feedback. This is the abstract section with limited wordcount, so we have summarized the findings in the abstract section, while the detailed explanation has been provided in the Results section of the manuscript (not abstract). However, we have explained within the given word count for better clarity. The terminology 'self-awareness' is the standard terminology proposed by the WHO self-care framework, which we have adapted in this study. Clean version: Line: 35 to 44.

3. Conclusion: Don’t use acronyms (line 54). The conclusion should explain the implications of the findings and their relevance. You have included recommendations, and these don’t clearly relate to the results.

Answer: Thank you for the comment. We have incorporated it accordingly. Clean version Line 47 to 52.

**Introduction**

4. Include a definition or explanation of ‘self-care’ by building on the WHO framework – eg self care is defined as xxx, which includes self-awareness, self-management… This is a considerable omission because the whole paper hangs on the practice self-care.

Answer: Thank you for highlighting this. we have incorporated the definition of self-care on WHO framework in the line 58 to 63 of clean version.

5. Line 94: I’m not sure the disability cards ensure’ inclusive development. Best use less definitive language, such as ‘To facilitate inclusive devt, the govt….’

Answer: Thank you for the comment. We have adjusted the sentence accordingly in the line 114 of the clean version.

6. Line 108: needs a full stop and a reference for the health issues

Answer: Thank you for highlighting this. I have incorporated it accordingly in the line 129 of the clean version.

7. When describing the situation in Nepal, can you include some data on Karnali so it builds the case for you doing your study there?

Answer: Thank you very much for the insights. We have incorporated it from line 107 to 113 in the clean version of manuscript.

8. Line 112-115. Without a definition of ‘self-care’ this sentence doesn’t make sense

Answer: Thank you for pinpointing this. We have included the definition of self-care from the line 56 to 59.

9. Line 116-118 – needs a reference

Answer: Thank you very much for the comment. We have included the references accordingly in the line 136.

10. Line 116-122: include your research objectives. You reference objectives in line 132 and you must include them explicitly

Answer: Thank you for highlighting this. I have included the objectives in the clean version of the manuscript from line 136 to 140.

**Methods and materials**

11. Line 127 – reference Braun and Clarke, and give a brief explanation of this approach

Answer: Thank you very much for the comment. We have kept the reference and have provided a brief explanation of this approach. Line 143 to 150 in the clean version of manuscript.

12. Line 128 - Include information about Karnali province

Answer: Thank you very much for the comment. It has been incorporated accordingly from the line 105 to 111 of the clean version.

**Participants and sampling:**

13. Include your definition of ‘physical disability.’ How did you determine this in your study (e.g. self-reported or from medical lists)?

Answer: we have included the definition of physical disability in the introduction from the line 93 to 96 in the clean version of manuscript. Physical disability was chosen as it has a high prevalence compared to other disabilities (according to the Census of Nepal 2021). Three organizations of persons with disabilities (OPD) (Disable Rehabilitation & Rural Development Organization (DARRDO) Nepal, Blind Women Association Nepal (BWAN) Surkhet Chapter, and Blind Youth Association Nepal (BYAN) Surkhet Chapter) facilitate the determination of potential participants. This has been described in Line 153 to 159 of the clean version of manuscript.

14.Line 130-133: ‘better explain their experiences according to the study’s objective’ introduces researcher bias as you could have excluded relevant participants. How did you attempt to manage that? How can you justify this approach?

Answer: Thank you very much for the comment. I have included the objective of the study, removing the researcher bias. Only individuals who voluntarily agreed to participate and provided informed consent to share their experiences were listed. Women with physical disabilities under 18 years of age were excluded, as marriage before 18 years is illegal in Nepal. Additionally, any participants who did not provide informed consent were excluded. Based on these criteria, the principal investigator finalized the list of participants. This has been incorporated from the line 161 to 165 of the clean version of manuscript.

15. Why was ‘married women’ important?

Answer: According to a 2022 report by The Rising Nepal, approximately 52% of child marriages occur in Karnali Province of Nepal. Thus, women of reproductive age (15-49 years) are mostly married in Karnali province thus, married women were taken as the study population.

16. It would be helpful if you specifically state what your inclusion/exclusion criteria is?

Answer: Thank you very much for your comment. We have specifically stated the inclusion and exclusion criteria as per your suggestion. This has been well explained in the Participants and Sampling subheading from the line 152 to 165 in the clean version of the manuscript.

17. Include a separate section on ethical considerations. You need to put a lot more information in about the ethical procedures you followed. This would include the informed consent process, which ethical board you sought and received ethical clearance from, where you conducted the interviews etc etc. Some of this is covered under ‘data collection and procedures’. You can look at published qualitative papers related to disability to see what should be included and the level of detail

Answer: Thank you very much for the insight. We have added a section on ethical consideration as suggested by the reviewer in the line 209 to 219 in the clean version of Manuscript.

18. Reference the supporting documents were relevant (eg topic guides)

Answer: Since it was made under the description made by Barun and Clarke and SAGE Handb Qual Res Des. 2022;(April):290–306, this has been referenced. Also, the interview guide and consent form have been submitted as supporting documents in the submission portal.

**Results**

19. Line 199-202 and Table 2 should go under methods

Answer: The codebook and explanation of sub-themes and themes are results/findings of the study, rather than the method. So, we have kept it in the results section only. Thank you for your feedback.

20. You use terms like ‘moderate’, ‘severe’ disability and severity of disability (line 262-263), but you have not explained what you mean by this and the criteria you used to define moderate in the methods. This introduces researcher bias. Either delete the reference to this throughout the results or explain the criterion in your methods.

Answer: Thank you very much for your comment. All the terms are described under the introduction section to remove any confusion or ambiguity. Line: 96 to 103 of the clean version of Manuscript.

21. Line 226-227 – change to ‘their unborn children’ as that is what the participant is referring to, not living children

Answer: Thank you very much for pinpointing it. We have corrected accordingly. Line 396 of the clean version of manuscript.

22. Line 242 – define menstrual underwear. What are these, are they bought, are the reusable? Line 252 – you refer to menstrual panties, period panty. Use one term consistently throughout. In your introduction, include menstrual underwear as a product used in Nepal

Answer: Thank you for the comment. As per your feedback, I have made the consistent terminology as “menstrual underwear”. Menstrual underwear or period underwear are absorbent underpants that have multiple layers of microfiber polyester. They look like regular underwear, but they’re designed to keep moisture away from the skin as they soak up menstrual blood. The fabric in period underwear contains a moisture-wicking fabric made up of thousands of small filaments. These fibers trap blood or other liquid to keep it from leaking onto the clothes. The outer layer usually includes nylon and Lycra, and is then finished with a liquid-repellent film. ( <https://www.webmd.com/women/period-underwear>). Not only in Nepal but however, over the world, menstrual underwear is one of the menstrual product that is worn in during the mensturation to absorb the menstrual blood.

23. Line 245 – cloths, not clothes

Answer: Thank you very much for the comment. We have included it accordingly in the line 278 of the clean version of the manuscript.

24. Line 281-285 – full stops are missing. Ensure you closely proofread your manuscript before submission

Answer: Thank you very much for highlighting this. All the grammars in the manuscript have been thoroughly checked, proofread and ensured.

25. In academic writing, you always need to define terms used. Eg you refer to ‘proper disposal’; that must be explained as ‘proper’ can be subjective. Always use references when giving definitions. This doc might be helpful: <https://www.unicef.org/documents/guidance-menstrual-health-and-hygiene>

Answer:Thank you for your feedback and this has been well noted.

26. Line 310: Give an example to make this sentence clear.

Answer: Thank you very much for the comment. We have provided specific example on this. Line 351 to 366 of the clean version of manuscript.

27. Line 410: ‘some of the young’ – define the age range as you are only interviewing adults

Answer: Thank you very much for the comment, we have included the age range
(18-20)years. The comparison on the age range led to use the word young despite being all of them adult. This has been incorporated in the line 436 of the clean version of manuscript.

**Discussion**

28. Include a discussion on the transferability of research findings to other settings

Answer: Thank you very much for the comment. This has been explained under the section strengths and limitation. Line: 646 to 661 of the clean version of manuscript.

29. Line 515. Have a separate section on the study’s strengths and limitations

Answer: Thank you very much for the comment. This has been explained under the section strengths and limitation. Line: 646 to 661 of the clean version of the manuscript.

30.Reflect on the influence of the researcher[s] on data, including a consideration of how the researcher(s) may have introduced bias to the results is included

Answer: Thank you very much for the comment. This has been explained under the section strengths and limitation. Line: 646 to 661 of the clean version of the manuscript.

Reviewer #2:

1. Abstract: Undefined acronym: FCHV

Answer: Thank you for highlighting this. We have replaced the word with community volunteers in the clean version of the manuscript. Line: 46 of the clean version of manuscript.

2. Results: The status of physical disability is mentioned with all the quotes, and age can also be mentioned for a better understanding of the background of the participants.

Answer: To maintain anonymity and not provide information that could breach the anonymity, the participant's age has not been included. This approach was also followed during the first-round review process, in accordance with the given instructions.
