# Supplementary material for: Understanding the menstrual health self-care practices and experiences among women with physical disabilities in rural Nepal: A qualitative study
Source: PLoS One. 2025 Dec 10;20(12):e0338109. doi: 10.1371/journal.pone.0338109 (PMC12694883; doi:10.1371/journal.pone.0338109)
Supplement: Attachment — Submitted filename: Response to reviewers_third.docx [file pone.0338109.s007.docx]

1. **How did you analyze your data (Data analysis method/software vs manual )**

Answer: Thank you for your comment. The transcriptions and translations of the data were conducted verbatim, manually. For the coding of the translated data, inductive coding was conducted using Dedoose as data management software. This has been clearly incorporated in lines 215 to 230 of the clean manuscript

2. **Is it a monthly or lifetime history you took. The women may use sanitary pad sometimes and rag clothes on another time. The recent one she may used can affect your finding? (ups and downs in life )**

Answer: We collected participants’ lifetime histories documenting the sanitation practices and pad use, and the reasons for it. The participants in this study used either a sanitary pad or used clothes as a pad. Depending on the preference of the pad, the research focused on sanitation practices, which have been documented in the result section, lines 271 to 306 of the clean manuscript.

3. **Which type of physical disabilities did you consider in the study and why????**

Answer The participants in the study included individuals with varying degrees of severity of disability, profound (persons who face difficulty to perform day-to day activities even with continuous support of others), severe disability (persons who requires others support continuously to perform personal activities and involve in social activities), moderate disability (persons who can regularly participate in daily and social activities if physical, environmental, educational barrier is ended and mild disability (persons who can regularly participate in daily and social activities if physical and environmental barriers is dismantled (Line:97 to 103) as they are available physical disabilities of the clean manuscript.

**Why only married women? Since all women who started menstruations can be parts of the study.**

Answer: Choosing married women as participants helps to understand the experiences of menstruation from adolescence to adulthood, thus providing a comprehensive understanding of issues. The age of married women in this study ranges from 18-, thus gathering evidence from diverse women. Furthermore, compared to unmarried women with physical disabilities, married women with physical disabilities find it convenient to talk culturally about tabooed topics such as menstrual hygiene and can provide in-depth information from existing social norms, practices, and challenges for menstrual health management

4. **Why only 1 FGD? What was your justification and how did you able to determine the idea saturation with a single FGD????**

Answer: The study adopts IDI and FGD as data collection methods. We conducted 21 in-depth interviews. The FGD with 10 participants further helped to triangulate and validate the findings from the IDIs and helped to add information to reach the saturation. We adopted the definition of saturation from Fusch et. al- Are we there yet, as stated in lines 205 to 209 of the method section of the clean manuscript.

5. **Its better to indicate what severe, moderate, and mild disability mean in method section and what is your reference to classify them in such category??**

Answer: Thank you so much for your feedback. Thus has been discussed in the line 164 to 173 of the clean manuscript.

6. **If severity matters, did you consider including them from different severity status??**

Answer: This study has included different severity status- severe and moderate disability (2 out of 4 category) as the participants from these two categories has differential experience, as the objective of the study was to understand how the physical disability influences the menstrual hygiene experiences and practices. Even though initially, women with mild physical disability was included, they were excluded from the study, in consultation with partner organization, as there was no differential experience or impact to women without disability. This has been explained in the line 149-151 of the clean manuscript.

7. **The way dta collected was bit confusing? Make it clear.**

Answer: Thank you for your feedback, necessary edits has been made to make it clear from line .176 to 202.

8. **You have cited similar information with different source line…145 and 225. (refe 28 vs 31)**

Answer: Thank you for flagging this. We have edited the reference section accordingly.

9. **Did you use the deductive or inductive approach while you generate the “Themes’’? and why?**

Answer: We used an inductive approach for coding and aggregating codes to form sub-themes. The sub-themes with similar characteristics were aggregated to form themes as explained in the line: 227-230 of the clean manuscript.

10. **Is your finding different from what is common among general population? Especially in terms of menstrual hygiene materials utilized, awareness level, and in terms of pain relieving approaches?**

Answer: There are few observations such as: self-testing which is body symptoms, self-management of menstrual pain particularly using home remedies, and mental distress, particularly before (pre-menstrual syndrome and premenstrual dysphoric syndrome) and during menstruation due to fear of seclusion and discrimination practiced during menstruation. Similarly, women with physical disabilities using cloth pads highlighted the tediousness and difficulty in self-management of menstrual hygiene due to a lack of water and sunlight during winter, limited information and choice of menstrual product, limited access to menstrual health related information are some of the differential impact among women with physical disabilities compared to general population. This paper presents existing ways of self-care in menstrual health management and proposes way forward on how strengthening self-care could help enhance good health and well-being of women. This has been well explained in detail in result and discussion section of the manuscript.

11. The recommendation should also some thing inline with your finding and applicable (should be to bring tangible change if applied)

Answer: Thank you for your suggestion. We have proposed recommendations to strengthen self-care in menstrual health management for the overall health and well-being of women and girls, aligning with the findings in the discussion section from line 594 to 638 of the clean manuscript.
